# Organic Acids of the Microbiological Post-Culture Medium as Substrates to be Used for Starch Modification

**DOI:** 10.3390/polym11030469

**Published:** 2019-03-12

**Authors:** Ewa Zdybel, Tomasz Zięba, Waldemar Rymowicz, Ewa Tomaszewska-Ciosk

**Affiliations:** 1 Department of Food Storage and Technology, Faculty of Food Science, Wroclaw University of Environmental and Life Sciences, 37 Chełmońskiego Street, 51-630 Wrocław, Poland; tomasz.zieba@upwr.edu.pl (T.Z.); ewa.tomaszewska-ciosk@upwr.edu.pl (E.T.-C.); 2 Department of Biotechnology and Food Microbiology, Faculty of Food Science, Wroclaw University of Environmental and Life Sciences, 37 Chełmońskiego Street, 51-630 Wrocław, Poland; waldemar.rymowicz@upwr.edu.pl

**Keywords:** esterified starch, organic acid, *Yarrowia lipolitica* yeast, microbiological post-culture media

## Abstract

This study aimed to identify the feasibility of producing highly-substituted starch esters via thermal modification of starch using a post-culture medium of *Yarrowia lipolitica* yeast. This manuscript describes a successful attempt at potato starch modification with a post-culture medium of *Yarrowia lipolitica* yeast with different concentrations of organic acids. Starch preparations produced by roasting (130 °C) and these produced by starch reaction with a synthetic acid mixture were compared in terms of the types and number of acid residues esterified with starch. The effectiveness of starch esterification was found to depend on medium composition and to be higher upon the use of a synthetic acid mixture. In addition, a higher reactivity with starch was demonstrated for citric acid than for α-ketoglutaric acid. The highly-substituted starch esters formed as a result of potato starch modification with post-culture medium were characterized by decreased values of thermal parameters of pasting characteristics, determined with a differential scanning calorimeter (DSC), and by compromised resistance to amylolysis. The intensity of these changes increased along with an increasing total percentage of starch ester substitution.

## 1. Introduction

Starch—which plays the role of a storage substance in plants—is produced on the industrial scale mainly from maize, rice, tapioca, and potatoes. In 2018, global starch production was estimated to reach 133.5 mln tons [1]. Most of starch material is modified with enzymatic, physical, or chemical methods to tailor its properties to specific industrial applications [2]. Physically- or enzymatically-modified starch may be used in the food industry without any limits. In turn, chemically-modified starch preparations are produced from starch isolated from starchy materials using acids, their anhydrides, or salts, as well as oxidative or alkylating agents. Such modified preparations of low-substituted starch serve as food additives, and their use is regulated by law [3]. Among a wide array of chemically-modified starch preparations, oxidized starch (E1404), monostarch phosphate (E1410), distarch phosphate (E1412), phosphorylated distarch phosphate (E1413), acetylated distarch phosphate (E1414), acetylated starch (E1420), acetylated distarch adipate (E1422), hydroxypropyl starch (E1440), hydroxypropyl distarch phosphate (E1442), sodium starch octenylsuccinate (E1450), acetylated oxidized starch (E1451), and aluminum starch octenylsuccinate (E1452) are permitted for use as food additives [4].

Chemical processes employed in food technology are those based on chemical reactions which require specified chemical substances and proceed without any biological factors [5]. It must be remembered, however, that during food manufacture the native plant starch enters into contact with other substances and under favorable conditions may undergo chemical transformations not classified as chemical operations in legal terms. These substances include acids which have a carboxyl group which may bind via ester bonds with the hydroxyl group of starch. Many works are available on both the methods of starch modification as well as the properties of produced esters. Starch heating with citric acid at temperatures exceeding 100 °C is described in the literature as a method used to produce crosslinked starch, i.e., starch whose two chains are bound with acid residues [6,7]. Other studies have shown the feasibility of producing esters of crosslinked starch by using crosslinked starch as a substrate in the esterification reaction [8].

Rich sources of organic acids include, among others, microbiological post-culture media deemed to be waste products after the production of acids by *Yarrowia lipolitica* yeast [9]. An exceptional trait of this yeast species is its capability to absorb carbon from agri-food waste and to produce high quantities of valuable metabolites, like citric acid, alpha-ketoglutaric acid, pyruvic acid, or succinic acid [10,11,12]. This method of acid production is more effective, less expensive, and ecologically safer compared to chemical methods. Once the biomass and yeast metabolites are isolated from the culture, a waste remains, namely, a post-culture medium which contains organic substances. Their management may contribute to the optimization of the use of substances produced by yeast as well as to the protection of the natural environment via reduction of the load of waste substances. The post-culture media contain organic acids, the composition and ratios of which depend on the yeast species, substrate, and culture conditions used [12]. The feasibility of using post-culture media, despite their varying composition, for starch esterification with no need for the earlier isolation of organic acids, may be of great technological and economic importance, and may offer an interesting alternative to the chemical operations used in the food industry. In addition, among ample research works describing possibilities of starch modification with various reagents, relatively few reports are available which address starch esterification using a mixture of acids, especially these of natural origin.

The major objective of this study was to identify the feasibility of producing highly-substituted starch esters during thermal modification of starch using a post-culture medium of *Yarrowia lipolitica* yeast containing various concentrations of organic acids, and then to determine changes in the thermal properties of the modified preparations as well as their susceptibility to amylolysis.

## 2. Materials and Methods

### 2.1. Materials

Potato starch produced by PPZ Niechlów, Poland, in 2017 was modified with four post-culture media of *Yarrowia lipolitica* yeast containing a mixture of carboxylic acids. The media were obtained from the Department of Food Biotechnology and Microbiology of the Wrocław University of Life Sciences, and their composition was as follows:

- ABK medium—α-ketoglutaric acid 26.5 g/L; citric acid 73.0 g/L
- α20K medium—α-ketoglutaric acid 55.7 g/L; pyruvic acids 3.7 g/L
- Y20K medium—α-ketoglutaric acid 51.5 g/L; citric acid 14.9 g/L; pyruvic acid 5.0 g/L
- X16M medium—citric acid 69.9 g/L; α-ketoglutaric acid 28.7 g/L

Concentrations of acids in these media were determined using the high performance liquid chromatography (HPLC) method (described in Section 2.3).

In addition, starch preparations were produced with aqueous solutions of the following acids (at ratios identical to those in the culture media): citric acid, α-ketoglutaric acid, and pyruvic acid (all of analytically pure grade); all acids were produced by Sigma (Steinheim, Germany).

### 2.2. Production of Starch Preparations

Starch preparations were produced according to the method used for starch citrate production described by Kapelko-Żeberska et al. [6]. Native potato starch was mixed with the post-culture media or with the synthetic mixture of acids at the following ratios: 20 g of citric acid (in the case of ABK and X16M media) per 100 g of starch dry matter or 20 g of α-ketoglutaric acid (in the case of α20K and Y20K media) per 100 g of starch dry matter. The resultant mixtures were air-dried at a temperature of 40 °C for 24 h, and then cooled and ground. The dried and disintegrated mixtures were roasted in a convective dryer (Memmert, Schwabach, Germany) at a temperature of 130 °C for 3 h. To remove the excess reagent, the roasted samples were rinsed with portions of 60% ethyl alcohol (Honeywell, Seelze, Germany) at an alcohol to sample ratio of 4:1. After ten-fold rinsing, the samples were poured into 60% ethanol and left covered at room temperature for 24 h. The entire rinsing cycle was repeated three times.

Starch modification with a synthetic mixture of carboxylic acids was conducted analogously to starch modification with acids of the post-culture media.

### 2.3. Determination of the Degree of Substitution

The degree of substitution was expressed in grams of acid residues per 100 g of starch preparation (substitution percentage). The content of acid residues esterified with starch was determined with HPLC (Thermo Fisher, Wien, Austria) on a Carbohydrate H^+^ column coupled with a UV detector (λ = 210 nm) and a refractometric detector at a temperature of 65 °C, with a liquid phase (25 nM trifluoroacetic acid) flow rate of 0.6 mL min^−1^. Compounds were identified based on chromatograms of standards of pure chemical substances at the Department of Food Biotechnology and Microbiology of the Wrocław University of Life Sciences [10,13].

To prepare the samples for HPLC analysis of acid residues, 2 g of starch preparation was treated with 100 mL of 0.5 M NaOH (Chempur, Piekary Śląskie, Poland) for 24 h under continuous stirring. Afterwards, 250 mL of concentrated ethyl alcohol (Honeywell, Seelze, Germany) was added and the solution was left at room temperature for 24 h. Next, the solution was filtrated and the filtrate obtained was concentrated to a volume of 50 mL. Thus, prepared solutions were determined for the content of acid residues. The analysis was conducted in triplicate.

### 2.4. Calculation of Effectiveness of Esterification Reaction

The substitution percentage was used to compute an esterification yield understood as the ratio of the obtained substitution percentage to the theoretical highest substitution percentage possible at the acid dose applied [14,15].

### 2.5. Characteristics of Phase Transitions

The characteristics of phase transitions were determined for the starch preparations produced with the post-culture media. Native starch and starch roasted without the modifying agent were used as reference samples. Phase transition characteristics was determined using the differential scanning calorimeter DSC 822 (Mettler Toledo, Schwerzenbach, Switzerland) in 100 µL aluminum crucibles with lids. Dry matter (10 mg) of the analyzed samples and water were weighed into a crucible at a ratio of 1:3. The crucible was covered with a lid, conditioned at room temperature for 30 min, and then placed in a calorimeter chamber at 25 °C and heated to 100 °C at a heating rate of 4 °C/min. The thermal curve plotted was used to determine the temperatures of the onset, maximum, and end of the phase transition, as well as the specific heat of transition. The analysis was conducted in triplicate [16].

### 2.6. Determination of the Susceptibility of Starch Preparations to Amylolytic Enzymes

Analyses were carried out for the starch preparations produced with the post-culture media. Native starch and starch roasted without the modifying agent were used as reference samples. A water suspension of starch preparations (0.72 g of preparation per 100 mL of solution) was heated at 100 °C for 5 min. The resultant paste was double diluted with an acetate buffer (pH = 4.3). Flasks with mixtures were put into a water bath with a temperature of 37 °C, and a mixture of α-amylase and glucoamylase enzymes (Sigma, Steinheim, Germany) was added. The dose of enzymes was adjusted so as to ensure complete saccharification after 20 min of native starch paste hydrolysis. The samples were incubated at 37 °C for 20 and 120 min. Afterwards, glucose content was determined in the samples using a glucose assay kit (BioSystem, Barcelona, Spain) which contained glucose oxidase and peroxidase. Following reaction with kit components, glucose contained in the samples formed a color complex, the concentration of which was measured spectrophotometrically (Cecil, Cambridge, England) at a wavelength of 500 nm and compared to a standard curve. Saccharification of starch preparations exposed to a mixture of amylolytic enzymes for 20 and 120 min was calculated and used to compute the percentage of fractions of rapidly-digestible starch (RDS), slowly-digestible starch (SDS), and starch resistant to amylolysis, in the starch preparations. The analysis was conducted in triplicate [6,17].

### 2.7. Statistical Analysis

Study results were subjected to statistical analysis using a Statistica 13.1 software package. Two-way analysis of variance was conducted. The significance of differences between mean values was determined with Duncan’s test at a significance level of α = 0.05.

## 3. Results and Discussion

In this study, an attempt has been made to modify potato starch using a post-culture medium of *Yarrowia lipolitica* yeast. This type of post-culture media management has been rarely addressed in research works.

**Figure 1:**
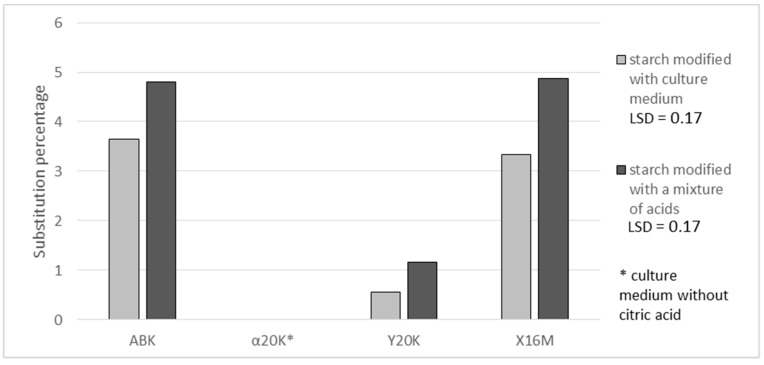
Effect of applied post-culture medium or a synthetic mixture of acids on the percentage of substitution of potato starch preparations with citric acid.

**Figure 2:**
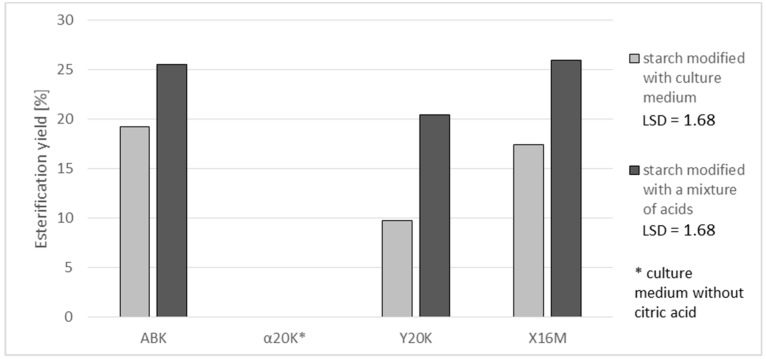
Effect of applied post-culture medium or a synthetic mixture of acids on the yield of starch esterification with citric acid.

Figure 1 present results of determinations of a substitution degree expressed in grams of citric acid residues in 100 g of the preparation. In the case of citric acid (Figure 1), the highest achieved substitution percentage was 4.9%. Comparable content of this acid was reported by Mei et al. [18], who used citric acid to esterify cassava starch; by Kapelko-Żeberska et al. [6], who by using the same method of esterification obtained preparations with 2.5 to 5.5 g of citric acid residues per 100 g of the preparation; and by Olsson et al. [19], who investigated the possibility of using starch citrates in biodegradable package materials. The degree of substitution with citric acid reached 3.6 and 3.3% in the case of preparations produced with the post culture media containing ca. 70 g of citric acid/L (ABK and X16M media) and 0.5% in those produced with the post-culture medium containing 15 g of citric acid/L (Y20K medium). A higher percentage substitution with citric acid was achieved when using a mixture of acids instead of the post-culture medium. This was probably due to the non-uniform composition of the medium, the components of which can bind organic acids. In the case of using a synthetic mixture of acids, starch had no competitors in its reactions with acids. The available literature provides no reports on the comparison of starch esterifications with natural mixtures and with analogous mixtures of pure acids. Hence, it is difficult to explicitly establish the reasons behind the higher degree of esterification achieved with the use of synthetic mixtures. This observed fact is interesting and needs further research. Additionally, the yield of the starch reaction with citric acid was higher with the use of the acid mixture. An analysis of reaction effectiveness allows for the conclusion that it is advisable to use higher amounts of citric acid in starch esterification reactions because this not only enables the production of preparations with a higher degree of esterification but also ensures that proportionally more acid takes part in the reaction with starch than in the case of a reagent containing less acid being used (Figure 2). Similar dependencies between reaction yield and substitution degree have been reported by other authors. For example, Song et al. [15] modified rice starch with succinic acid anhydride and achieved an increase in reaction yield along with an increasing degree of substitution. Khalil et al. [14] obtained the same dependency when modifying maize starch.

**Figure 3:**
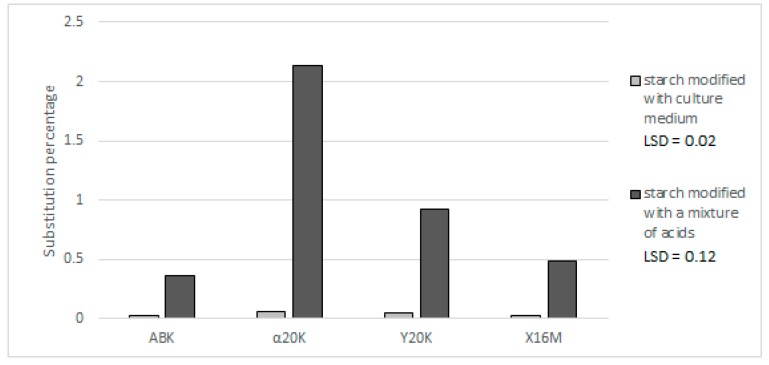
Effect of applied post-culture medium or a synthetic mixture of acids on the percentage of substitution of potato starch preparations with α-ketoglutaric acid.

**Figure 4:**
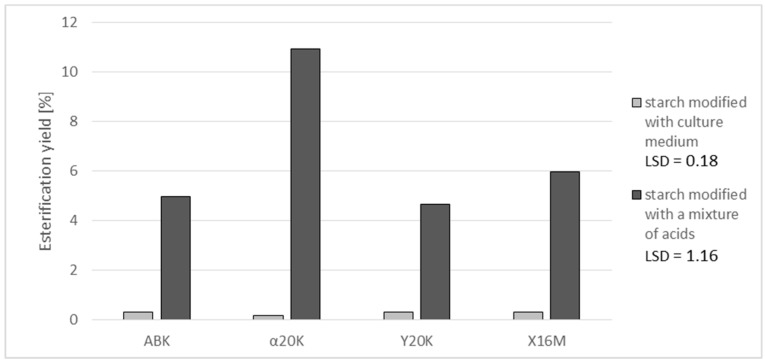
Effect of applied post-culture medium or a synthetic mixture of acids on the yield of starch esterification with α-ketoglutaric acid.

Lower affinity to starch was observed for alpha-ketoglutaric acid residues compared to citric acid. No studies are available in the literature that describe the possibility of starch modification with α-ketoglutaric acid and the properties of starch esters with this acid. In all preparations produced with the post-culture media, the percentage content of alpha-ketoglutaric acid was below 0.06% (Figure 3). Additionally, in the preparations produced with the acid mixture, its content ranged from 0.35% in the case of the ABK medium to 2.13% in the case of the α20K medium. It should be emphasized that the α20K and Y20K media, having similar concentrations of α-ketoglutaric acid, allowed for the production of preparations with significantly different contents of this acid (3.13 and 0.92%, respectively). It is most likely that starch esterification with residues of α-ketoglutaric acid is most effective when pure α-ketoglutaric serves as a substrate. In addition to α-ketoglutaric acid, the Y20K medium contained citric acid, which was the probable cause of the reduction in the effectiveness of the starch reaction with α-ketoglutaric acid (Figure 4). A similar observation was made for the culture media, as their non-uniform composition impaired reactions of starch chains with α-ketoglutaric acid. None of the produced preparations contained pyruvic acid, despite its presence in the post-culture media and synthetic acid mixtures. This was likely due to its too low concentration in the medium and in the mixture, and—as in the case of α-ketoglutaric acid—its low susceptibility to esterification with starch.

Results obtained for the substitution percentages were reflected in results of the thermal analysis (Table 1). Starch preparations with a higher substitution degree were characterized by the most decreased temperatures of the onset, end, and maximum of the phase transition, and by the lowest specific heat of the phase transition compared to native starch and to roasted native starch. Other authors have also reported decreases in values of thermal parameters of pasting as a result of starch chain substitution with acid residues [20,21]. The smallest changes in the discussed values were observed in the preparation modified with the α20K medium, which had the lowest substitution percentage.

**Figure 5:**
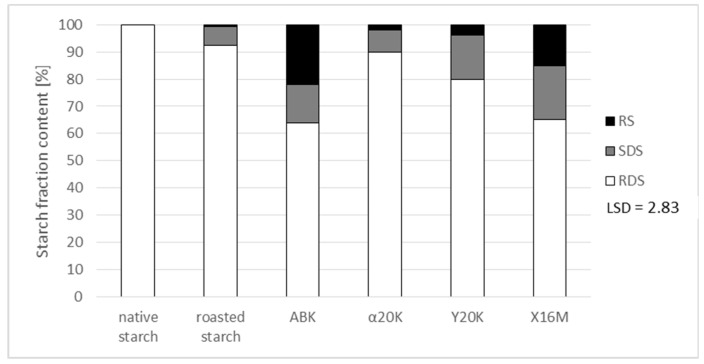
Contents of rapidly-digestible starch (RDS), slowly-digestible starch (SDS) and resistant starch (RS) fractions in starch preparations.

Preparations produced upon starch modification with the post-culture media were also analyzed for their susceptibility to amylolysis (Figure 5). This susceptibility was also determined as being affected by the roasting process. According to literature data [22], roasting induces thermolysis of starch chains, which is followed by the attachment of resultant glucose and short-chain dextrins non-specific for starch with bonds at carbon atoms in positions 2 and 3 of each glucose unit. These bonds are not hydrolyzed by amylases, and the attached dextrins hinder the access of enzymes to the starch chain. This effect manifested itself through the formation of small amounts of resistant starch (RS) and slowly-digestible starch (SDS) during native starch roasting. In chemically-modified starches, the attached substituents also decrease susceptibility to amylolysis [3,16,21]. According to literature data, starch esters with citric acid are characterized by reduced susceptibility to amylolysis [6]. The most decreased susceptibility to the action of enzymes and the highest content of the resistant starch fraction were determined in the preparations produced with the culture medium which had a high concentration of citric acid and which was characterized by the highest percentage of substitution with this acid. Furthermore, no significant increase in resistance was observed in preparations which were free of citric acid residues and in those which had a low substitution degree (0.03%) with α-ketoglutaric acid, compared to roasted starch. Preparations with reduced susceptibility to amylolysis may be used as health-promoting food additives [6].

## 4. Conclusions

1. In this study, potato starch modification with medium remaining after a culture of *Yarrowia lipolityca* yeast was observed to result in starch esterification with residues of organic acids contained in the medium, the effectiveness of which depends on the composition of the medium.
2. Esterification effectiveness after the use of the post-culture medium was seen to be lower than that observed after the use of a synthetic mixture of acids.
3. When the post-culture media and acid mixture were used, higher susceptibility to esterification with starch was noted for citric acid than for α-ketoglutaric acid.
4. Highly-substituted starch esters produced via potato starch modification with *Yarrowia lipolytica* post-culture medium were characterized by modified—respectively to the substitution degree—DSC pasting characteristics and resistance to amylolysis compared to native starch.

## Figures and Tables

**Table 1:** Thermal analysis of starch preparations produced with post-culture media.

Preparation	Temperature of Onset of Phase Transition (°C)	Temperature of Maximum of Phase Transition (°C)	Temperature of End of Phase Transition (°C)	Specific Heat of Phase Transition (J/g)
Native starch	62.63	66.99	73.38	13.15
Roasted native starch	54.71	61.19	69.83	11.79
ABK	38.06	43.22	50.18	1.51
α20K	44.43	53.61	64.38	10.43
Y20K	39.94	48.55	60.38	8.19
X16M	39.14	47.49	56.35	2.31
LSD	0.64	0.50	1.22	0.33
